# Hydroxytyrosol Protects against Myocardial Ischemia/Reperfusion Injury through a PI3K/Akt-Dependent Mechanism

**DOI:** 10.1155/2016/1232103

**Published:** 2016-02-04

**Authors:** Ying-hao Pei, Jiao Chen, Liang Xie, Xiao-min Cai, Run-Hua Yang, Xing Wang, Jian-bin Gong

**Affiliations:** ^1^Department of Intensive Care Unit, Jiangsu Provincial Hospital of Traditional Chinese Medicine, The Affiliated Hospital of Nanjing University of Traditional Chinese Medicine, 155 Han Zhong Road, Nanjing 210029, China; ^2^Department of Intensive Care Unit, Nanjing Integrated Traditional Chinese and Western Medicine Hospital, Nanjing University of Traditional Chinese Medicine, 179 Xiao Lin-Wei, Nanjing 210014, China; ^3^Department of Cardiology, Jinling Hospital, Nanjing University, School of Medicine, Nanjing 210002, China

## Abstract

*Objective*. To investigate the effects and mechanisms of hydroxytyrosol (HT) during the pathogenesis of myocardial ischemia reperfusion (I/R) in rat hearts.* Methods*. The rats were randomized into five groups: sham group, I/R group, HT+I/R group, HT+LY294002+I/R group, and LY+I/R group. Myocardial infarct size, markers of oxidative stress, extent of myocardial apoptosis, echocardiographically assessed cardiac function, and expression of Akt and GSK 3*β* were measured in each group.* Results*. Prereperfusion administration of HT was associated with a significantly smaller area of myocardial infarction and remarkably decreased level of myocardial apoptosis and necrosis, as evidenced by a lower apoptotic index, reduced cleaved caspase-3, and the serum activities of lactate dehydrogenase and creatinine kinase MB. Moreover, HT also attenuated the impairment of cardiac systolic function. However, cotreatment with LY294002 and HT completely abolished the above cardioprotective effects of HT. A subsequent mechanistic study revealed that the cardioprotective effects of HT during the process of I/R of the myocardium were dependent on the activation of the Akt/GSK3*β* pathway.* Conclusion*. Pretreatment with HT may have antiapoptotic and cardioprotective effects against myocardial I/R injury, and these effects seem to be related to the activation of the Akt/GSK3*β* pathway in the myocardium.

## 1. Introduction

Acute myocardial infarction (AMI) has become a major cause of mortality and morbidity worldwide. As the most successful therapeutic strategy, pharmacological or mechanical restoration of coronary blood flow has been established to be essential for preserving viable myocardium after AMI [1]. Although reperfusion therapy brings a new hope by reducing myocardial damage, reperfusion itself may induce a localized oxidative burst and regional inflammatory response, which causes cell damage and even death. This pathophysiologic process has been defined as ischemia/reperfusion injury (I/R injury) [2]. Numerous interventions and agents have been developed as prophylaxis for cardiac I/R injury, such as erythropoietin [3], alpha-lipoic acid [4], and nesfatin-1 [5]. However, translation of these strategies and agents to the clinical setting has not been satisfying. To improve clinical outcomes in AMI, it is of pivotal importance to develop new pharmacological agents for the prevention of myocardial I/R injury.

Olive oil is becoming more important in humans' daily diet due to its potential beneficial effects on health, particularly in the prevention of cardiovascular diseases and some cancers. Accumulating evidence suggests that most of the benefits of olive oil are mediated by its phenolic antioxidants. Hydroxytyrosol (3,4-dihydroxyphenylethanol, HT), the most active polyphenolic compound of olive oil and a potent scavenger of several free radical species, exhibits a protective action for cells against oxidative stress [6]. It has been demonstrated that HT is more active than synthetic antioxidants and may induce antioxidant enzymes to increase the endogenous defense system, thereby exerting an antioxidative stress effect in both* in vivo* and* in vitro* studies [7–9]. More importantly, HT has been indicated to exert protective effects against hepatic I/R injury [10]. However, to the best of our knowledge, it is still unknown whether HT has a protective effect against cardiac I/R injury.

Phosphoinositide 3-kinases (PI3K) and their downstream target serine/threonine kinase Akt are a conserved family of signal transduction enzymes that are involved in regulating cellular activation, inflammatory responses, and apoptosis [11]. Previous studies indicated that the PI3K/Akt signaling pathway may be an endogenous negative feedback regulator that forms a compensatory mechanism to limit proinflammatory and apoptotic events in response to harmful stimuli [12]. Activation of PI3K/Akt-dependent signaling has been shown to prevent cardiac myocyte apoptosis and attenuate myocardial I/R injury [13]. However, whether the PI3K/Akt pathway mediates the cardioprotective effects of HT has not been determined.

Therefore, in the present study, we aimed to investigate (1) whether HT protects rat myocardium from I/R injury* in vivo* in a rat model and (2) the possible role of PI3K/Akt signaling in the protective effects of HT against myocardial I/R injury.

## 2. Methods

### 2.1. Experimental Animals and Reagents

Adult male Sprague-Dawley rats, weighing 250–300 g, were purchased from the Animal Center of Jinling Hospital (Nanjing, China) and maintained for at least 1 week in a specific pathogen-free animal facility to allow adjustment to the environment. The animals were housed in groups of eight and maintained in controlled conditions of temperature (22 ± 2°C) and humidity (60–70%) and under a 12 h light-dark cycle (lights on 07:00 AM). Food and water were available ad libitum. All procedures were performed in accordance with the guidelines of the National Research Council's Guide for the Humane Care and Use of Laboratory Animals.

HT and LY294002, a PI3K inhibitor, were purchased from Sigma-Aldrich (St. Louis, MO, USA). The purity of HT (>98%) was determined by high-performance liquid chromatography. LY294002 was dissolved in dimethyl sulfoxide (DMSO; 0.002%). Anti-phospho-Akt (Ser473, Anti-pAkt), anti-Akt, anti-glycogen synthase kinase (GSK) 3*β*, anti-phospho-GSK3*β* (Ser9, anti-pGSK3*β*), anti-cleaved caspase-3, and anti-caspase-3 antibody were purchased from Sigma-Aldrich.

### 2.2. In Vivo Myocardial I/R Model

Rats were anesthetized by an intraperitoneal injection (*ip*) of 100 mg/kg ketamine. The rats were intubated and mechanically ventilated with room air using a rodent respirator (DV-2000, Shanghai Jia Peng Technology Co., Ltd., China). A limb lead II electrocardiogram (ECG) was recorded. An oscilloscope electrocardiogram monitor was used to display the electrocardiogram continuously throughout the procedure. A left thoracotomy was carried out to expose the hearts. Ligation of the left anterior descending (LAD) artery was performed 2-3 mm from its origin between the pulmonary artery conus and the left atrium using a 6-0 silk Prolene suture. A small vinyl tube was placed on top of the vessel to form a snare for reversible coronary occlusion. Myocardial cyanosis and ECG evidence of injury were used to confirm successful occlusion of the LAD artery. The heart was subjected to regional ischemia for 30 min, followed by coronary reperfusion through release of the slipknot.

### 2.3. Experimental Groups

Preliminary experiments testing three doses of HT (1, 5, and 20 mg/kg) showed that 20 mg/kg HT was the optimal dose based on the myocardial infarction size (49.5 ± 6.7%, 47.6 ± 7.9%, and 36.6 ± 3.9%, resp.). In the following study, a total of 100 rats were randomized to five groups: (1) sham group: rats subjected to the surgical procedures without coronary occlusion; (2) I/R group: 30 min coronary occlusion followed by 3 h reperfusion, normal saline* ip *5 min before reperfusion; (3) HT+I/R group: 20 mg/kg HT diluted in normal saline* ip* 5 min before reperfusion; (4) HT+LY+I/R group: 20 mg/kg HT diluted in normal saline* ip* and 0.3 mg/kg LY294002* ip* 5 min before reperfusion; and (5) LY+I/R group: 0.3 mg/kg LY294002* ip* 5 min before reperfusion.

### 2.4. Measurement of Myocardial Infarction Size

Infarction size was evaluated by Evans Blue/triphenyl tetrazolium chloride (TTC) staining as described previously [14]. Briefly, at the end of reperfusion, the LAD was religated and 2 mL Evans Blue (2%, Sigma-Aldrich) was injected intravenously to denote the area at risk. The heart was isolated and then frozen and sectioned perpendicular to the long axis (1.5 mm thick) up to the area of ligation. The slices were incubated in 1% TTC solution at 37°C for 20 min to visualize the infarcted area and transferred to 10% neutral buffer formalin overnight at room temperature to stabilize the staining. The Evans Blue-stained area represented the area not at risk. The area stained red by TTC represented ischemic but viable tissue. The infarcted myocardium was not stained by either TTC or Evans Blue and was paler than the TTC-stained area. The infarct area (IA) and area at risk (AAR) were determined by computerized planimetry using Image Pro Plus software (Media Cybernetics, Rockville, MD, USA).

### 2.5. Determination of Myocardial Apoptosis

Myocardial apoptosis was determined by terminal deoxynucleotidyl transferase-mediated dUTP-biotin nick end labeling (TUNEL) staining and myocardial caspase-3 activity after 3 h of reperfusion. TUNEL staining was performed according to the manufacturer's protocol (Roche). The apoptotic index (AI) was determined as the number of TUNEL-positive nuclei divided by the total number of nuclei stained in each field at a magnification of ×400. Cleaved caspase-3 levels were examined via western blot analysis.

### 2.6. Determination of Cardiac Function

Five rats from each group were used for cardiac function determination. To avoid interference in the acoustic signal by residual air trapped inside the chest cavity, echocardiography was conducted after 72 h of reperfusion, by which time most of the residual air had been absorbed. Baseline echocardiography was obtained 30 min before surgery. M-mode echocardiography was used to evaluate the cardiac dimensions and function by an echocardiography system with a 15 MHz linear transducer (Visual Sonics Vevo 2100, Canada). All of these measurements were performed in a blinded manner.

### 2.7. Western Blot

Cytoplasmic proteins were prepared from heart tissues, and immunoblots were performed as previously described [4]. In brief, tissue proteins were obtained from the heart 3 h after reperfusion in each group and were lysed in ice-cold extraction buffer containing protease inhibitor cocktail for 30 min. The whole lysates were then centrifuged at 12,000 ×g for 30 min, and the protein concentration in the supernatant was determined using a modified Bradford assay (Bio-Rad Laboratories, Hercules, CA, USA). The proteins were separated by electrophoresis via sodium dodecyl sulfate- (SDS-) polyacrylamide gel electrophoresis (PAGE), transferred to nitrocellulose membranes, and probed with primary antibodies against Akt, phosphor-Akt (Ser-473), GSK-3*β*, and phosphor-GSK-3*β* (Ser-9) (Sigma-Aldrich), followed by incubation with peroxidase-conjugated secondary antibodies (Sigma-Aldrich). The signals were detected with the enhanced chemiluminescence (ECL) system (Amersham Pharmacia).

### 2.8. Measurements of LDH and CK-MB

After 3 h of reperfusion, blood samples were collected from the right ventricle and centrifuged at 3000 ×g for 10 min to isolate serum. Myocardial cellular damage was evaluated by measuring lactate dehydrogenase (LDH) and creatinine kinase MB (CK-MB) activity in plasma using commercially available assay kits (Sigma-Aldrich).

### 2.9. Measurement of Oxidative Stress Markers

Superoxide dismutase (SOD) activity and malondialdehyde (MDA) content were used as indicators of oxygen free radical and lipid superoxide levels. The content of MDA and level of SOD in the tissue from the at-risk area of the left ventricle were measured using commercially available assay kits according to the manufacturer's instruction.

Mitochondrial and cytoplasmic SOD activities were measured independently. Isolation of myocardial mitochondria and cytoplasm was performed as previously described [15].

### 2.10. Statistical Analyses

Data are presented as mean ± standard error (SE). Statistical significance in multiple comparisons was evaluated by one-way analysis of variance using SPSS version 20.0 software (SPSS, Chicago, IL, USA). Values were considered statistically significant at a probability (*P*) of <0.05.

## 3. Results

### 3.1. Myocardial Infarction Size

**Figure 1:**
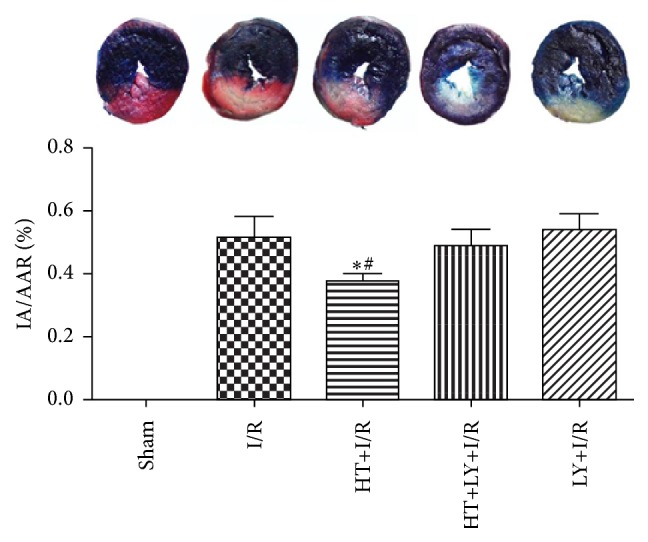
Effects of HT and LY294002 (LY) on the area of myocardial infarction (*n* = 4 for each group). Representative images of TTC and Evans blue dye staining of sections of the heart. Blue, nonrisk area; gray, IA; gray + brick red, AAR. The bar graph represents IA expressed as the percentage of the AAR. Data are presented as mean ± SE. ^*∗*^ *P* < 0.05 versus I/R group. ^#^ *P* < 0.05 versus HT+LY+I/R group.

Representative images of the TTC and Evans Blue staining for rats in each group were shown in Figure 1. Compared with rats in I/R group, HT administration leads to a significant reduction in the area of myocardial infarction induced by I/R injury (38.6 ± 3.1% versus 51.3 ± 7.7%, *P* < 0.05). However, the protective effect of HT on the size of the myocardial infarcted area was abolished by LY294002 treatment (HT+LY+I/R group: 48.8 ± 5.6%, LY+I/R group: 53.2 ± 6.7% versus I/R group: 51.3 ± 7.7%, *P* > 0.05; Figure 1).

**Figure 2:**
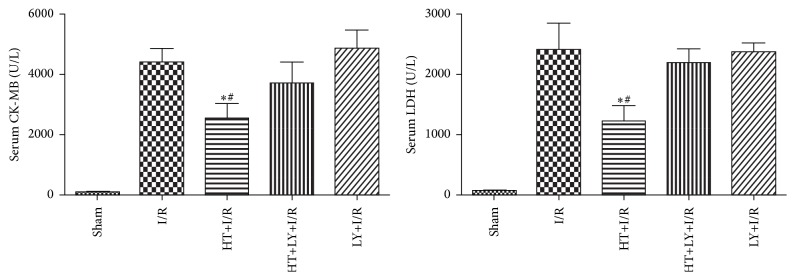
Effects of HT on serum levels of CK-MB and LDH (*n* = 20 for each group). ^*∗*^ *P* < 0.05 versus I/R group. ^#^ *P* < 0.05 versus HT+LY+I/R group.

Serum levels of CK-MB and LDH were also served as indicators for myocardial injury evaluation in our study. Both the plasma CK-MB and LDH levels were significantly increased in rats of the I/R group compared with those in the sham group. Consistently, HT treatment reduced the levels of CK-MB and LDH as compared with those in the rats of the I/R group (CK-MB: 2362.6 ± 462.4 U/L versus 4339.2 ± 404.3 U/L, LDH: 1101.2 ± 303.7 U/L versus 2548.9 ± 394.8 U/L, resp.; both *P* < 0.05). However, LY294002 administration eliminated the reducing effect of HT on both CK-MB and LDH (CK-MB: 2362.6 ± 462.4 U/L versus 3667.3 ± 584.3 U/L, LDH: 1101.2 ± 303.7 U/L versus 2143.2 ± 224.4 U/L, resp.; both *P* < 0.05; Figure 2).

### 3.2. MDA Content and SOD Activity

**Figure 3:**
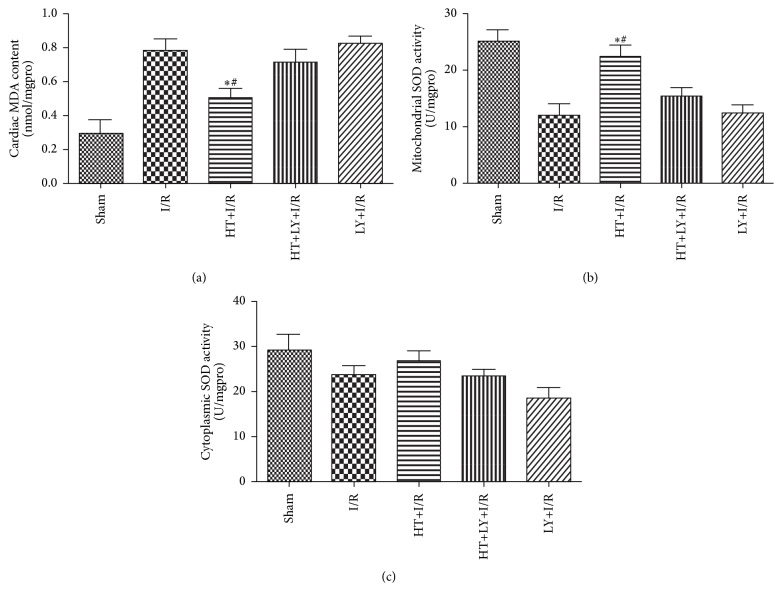
Effects of HT on myocardial levels of MDA (a) and SOD (mitochondrial: (b) and cytoplasmic: (c)) (*n* = 4 for each group). ^*∗*^ *P* < 0.05 versus I/R group. ^#^ *P* < 0.05 versus HT+LY+I/R group.

In comparison with that in the IR group, a significant reduction in the content of MDA (0.523 ± 0.078 nmol/mgpro versus 0.792 ± 0.096 nmol/mgpro, *P* < 0.05) and an increase in mitochondrial SOD activity (23.3 ± 2.2 U/mgpro versus 11.8 ± 2.0 U/mgpro, *P* < 0.05) were observed for rats in the HT+I/R group (*P* < 0.05). However, addition of LY294002 attenuated the changes in MDA content and SOD activity induced by HT treatment (MDA: 0.523 ± 0.078 nmol/mgpro versus 0.702 ± 0.052 nmol/mgpro, SOD: 48.3 ± 3.4 U/mgpro versus 40.3 ± 6.8 U/mgpro, both *P* < 0.05; Figure 3).

### 3.3. Myocardial Apoptosis

**Figure 4:**
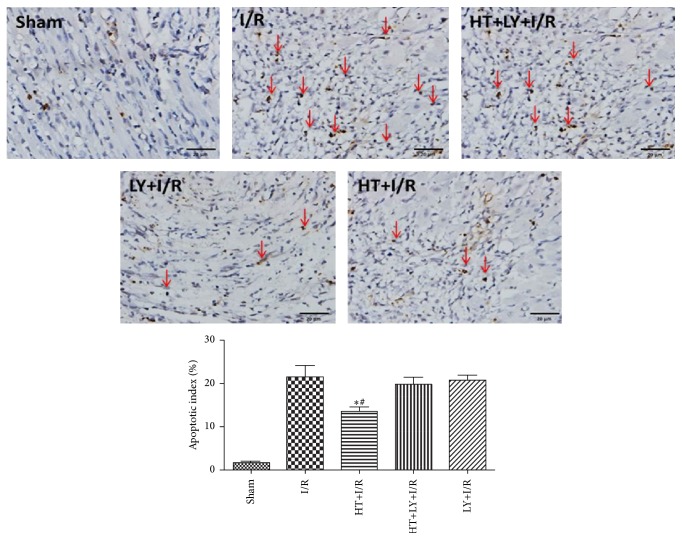
HT treatment attenuates cardiomyocyte apoptosis following myocardial I/R (*n* = 4 for each group). Hearts were harvested and sectioned for analysis of apoptosis by the TUNEL assay. Blue staining indicates the nucleus of each cell, and the dark brown staining indicates apoptotic cardiomyocytes, which are marked by red arrows. The bar graph shows the percentage of apoptotic cells. ^*∗*^ *P* < 0.05 versus I/R group. ^#^ *P* < 0.05 versus HT+LY+I/R group.

**Figure 5:**
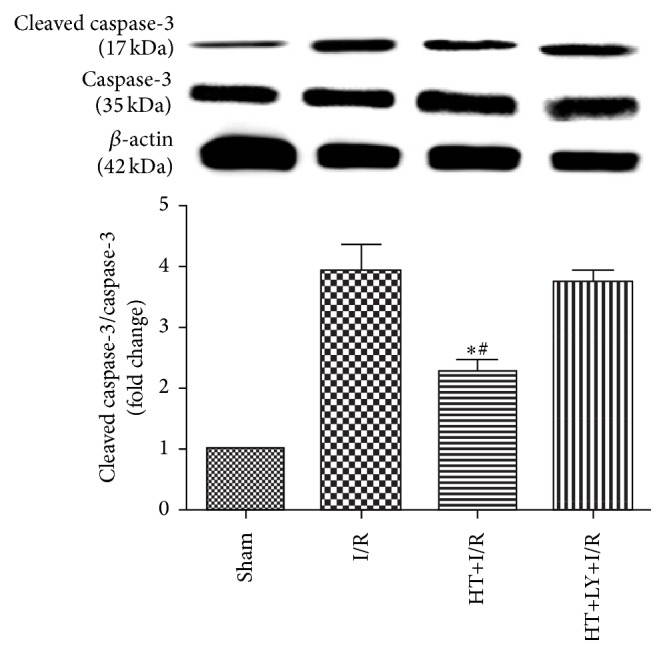
HT decreased cleaved caspase-3 expression as compared with expression levels in the I/R group and the HT+LY+I/R group (*n* = 4 for each group). Data are presented as means and SEM. ^*∗*^ *P* < 0.05 versus I/R group. ^#^ *P* < 0.05 versus HT+LY+I/R group.

The detection of TUNEL positivity is used to evaluate the levels of myocardial apoptosis. The AIs in the myocardium of rats of the HT+I/R group were significantly lower than those in rats in the I/R group (13.3 ± 2.1% versus 22.1 ± 4.3%, *P* < 0.05), but this antiapoptotic effect of HT could be attenuated by LY294002 administration (19.4 ± 2.2%, *P* < 0.05 compared with the HT+I/R group; Figure 4). Western blot analysis demonstrated lower cleaved caspase-3 levels in the myocardium of rats from the HT+I/R group compared with those in both the I/R group and HT+LY+I/R group (*P* < 0.05; Figure 5).

### 3.4. Cardiac Function

The heart rates of the rats in the sham group, I/R group, HT+I/R group, HT+LY+I/R group, and LY+I/R group before surgery were 288.4 ± 11.2 bpm, 292.1 ± 8.2 bpm, 289.5 ± 6.1 bpm, 293.6 ± 9.3 bpm, and 296.7 ± 4.8 bpm, respectively. These heart rates did not differ significantly after coronary ischemia (295.0 ± 5.6 bpm, 312.1 ± 12.6 bpm, 304.5 ± 9.1 bpm, 313.2 ± 14.5 bpm, and 316.5 ± 12.5 bpm, resp., all *P* > 0.05 compared with data before surgery) or reperfusion (292.5 ± 13.3 bpm, 293.9 ± 10.3 bpm, 283.7 ± 15.3 bpm, 298.6 ± 16.3 bpm, and 300.1 ± 9.2 bpm, resp., both *P* > 0.05 compared with data before surgery).

**Figure 6:**
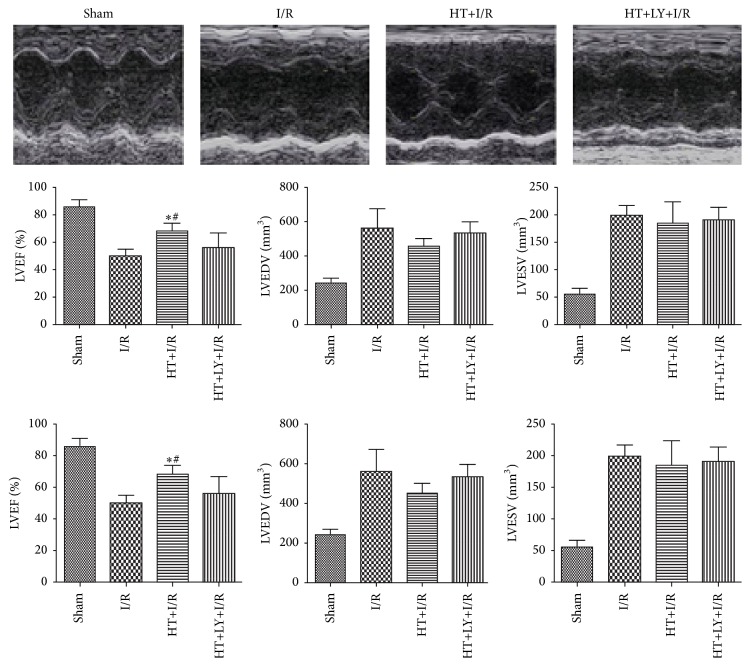
Echocardiography was conducted at two time points: 30 min before surgery to obtain baseline data and 72 h after reperfusion (*n* = 4 for each group). Representative images of echocardiographic examination showed that the HT group has better left ventricular free wall motion compared with the I/R group and the HT+LY+I/R group. HT administration significantly enhanced LVEF as compared with that in the I/R group and HT+LY+I/R group. ^*∗*^ *P* < 0.05 versus I/R group. ^#^ *P* < 0.05 versus HT+LY+I/R group.

Echocardiography was conducted at two time points: 30 min before surgery to obtain baseline data and 72 h after reperfusion. No significant difference in the baseline echocardiographic evaluation was observed among the groups (data not shown). I/R injury impaired cardiac systolic function, as evidenced by a significant reduction in left ventricular ejection fraction (LVEF; 48.9 ± 3.7% versus 83.6 ± 4.7%, *P* < 0.05), an increase in left ventricular end-systolic volume (LVESV; 587.5 ± 84.5 mm^3^ versus 243.3 ± 22.8 mm^3^, *P* < 0.05), and an increase in left ventricular end-diastolic volume (LVEDV; 203.5 ± 22.5 mm^3^ versus 53.8 ± 8.7 mm^3^, *P* < 0.05) in rats in the I/R group compared with those in the sham group. HT treatment restored LVEF after I/R (68.2 ± 5.6% versus 48.9 ± 3.7%, *P* < 0.05), whereas LY294002 could prevent this cardiac protective effect of HT for systolic function (53.8 ± 7.7%, *P* < 0.05 compared with the HT+I/R group). The effect of HT on the changes in LVEDD and LVESD was not significant compared with those observed in the I/R group or the HT+LY+I/R group (*P* > 0.05; Figure 6).

### 3.5. Western Blot Analysis

**Figure 7:**
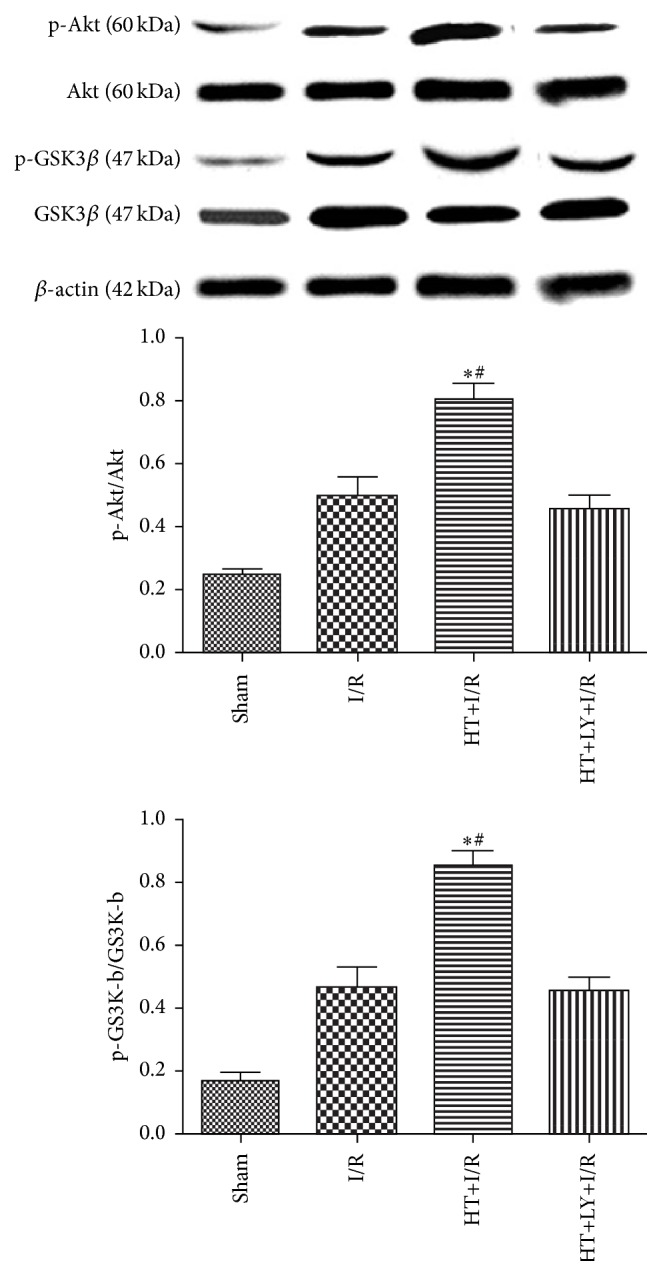
HT increased the levels of phosphorylated Akt and phosphorylated GSK-3*β* in the myocardium (*n* = 4 for each group). Phosphoinositide 3-kinase inhibition attenuates HT-induced increases in phospho-Akt and phospho-GSK3*β*. The levels of phospho-Akt and phospho-GSK-3*β* were examined by western blot analysis with specific antibodies. ^*∗*^ *P* < 0.05 versus I/R group. ^#^ *P* < 0.05 versus HT+LY+I/R group.

As shown in Figure 7, HT treatment significantly increased the myocardial levels of pAkt in rats in the I/R group compared with those in untreated I/R hearts (0.77 ± 0.04 versus 0.51 ± 0.06, *P* < 0.05). However, administration of LY294002, an inhibitor of PI3K, could significantly attenuate the HT-induced upregulation of myocardial pAkt in rats in the I/R group (0.47 ± 0.03, *P* < 0.05 compared with the HT+I/R group). Moreover, HT treatment significantly increased the levels of pGSK3*β*, a downstream kinase of Akt, in the myocardium compared with levels in untreated I/R hearts (0.82 ± 0.08 versus 0.47 ± 0.08, *P* < 0.05). However, administration of the PI3K inhibitor LY294002 could significantly prevent the HT-induced enhancement of phosphorylation of GSK3*β* in the I/R myocardium (0.42 ± 0.03, *P* < 0.05 compared with the HT+I/R group).

## 4. Discussion

In this study, we evaluated the potential therapeutic effects of HT in a model of rat I/R injury induced by ligation of the LAD. Moreover, we also explored whether regulation of the PI3K/Akt pathway, an important molecular signaling pathway in the pathogenesis of I/R injury, was involved. Our results showed that I/R injury could lead to severe myocardial necrosis and apoptosis, oxidative stress damage, and eventually myocardial infarction and cardiac dysfunction. Notably, pretreatment with 20 mg/kg HT had antioxidation and antiapoptotic effects, thereby significantly reducing the myocardial infarction size and preserving cardiac function. Activation of the PI3K/Akt pathway also was observed after treatment with HT. Moreover, all of the above therapeutic effects of Ht were abolished by administration of a PI3K inhibitor, suggesting that the benefits of HT for myocardial I/R injury are dependent on the regulation of PI3K/Akt pathways. To the best of our knowledge, this is the first study to demonstrate the protective effects of HT in myocardial I/R injury.

HT has been proven to exert antioxidant activity both* in vitro* and* in vivo*, which is probably related to its free radical scavenging activity [16–18]. After myocardial I/R injury in the myocardial tissue, it was revealed that increased levels of inflammatory cytokines such as TNF-*α*, IL-6, and IL-10 [19] and HT were able to influence many of these cytokines profiles [20–22]. Inflammatory damage has been shown to be a characteristic pathologic process in myocardial I/R injury and is associated with the accumulation of reactive oxygen species (ROS). Our study showed that HT pretreatment could alleviate oxidative stress during myocardial I/R injury at least by decreasing the content of MDA and increasing mitochondrial SOD activity in the myocardium. MDA is a product of cell membrane lipid peroxidation [23]. SOD is an oxygen radical scavenger that can protect cells against oxidative damage by converting superoxide anion radicals that occur in the upper stream of the reactive oxygen metabolism cascade [24]. Both of these enzymes play important roles in oxidative stress during I/R injury [25]. It is noteworthy that HT pretreatment can enhance mitochondrial SOD activities but not cytoplasmic SOD activities, which may be explained by the possible selective effect of HT on differentially distributed SOD activities. The potential underlying mechanism requires further investigation.

Cardiomyocyte apoptosis plays a key role in the development of myocardial infarction and cardiac dysfunction after ischemia [26]. Oxidative stress is one of the major stimuli of myocardial apoptosis [27]. In our study, the results showed that HT significantly reduced myocardial apoptosis, which was also consistent with oxidant stress and infarction size analysis. Because activation of the PI3K/Akt-dependent pathway has been considered to be associated with protection of cardiomyocytes from I/R injury and inhibition of I/R-induced cardiomyocyte apoptosis [28], we hypothesized that activation of PI3K/Akt activity in HT-treated rats may be responsible for the cardioprotection against I/R injury. We observed that the levels of phosphorylated Akt in the myocardium of HT-pretreated rats were higher than those in untreated rats. More importantly, by using the PI3K inhibitor LY294002, we found that pharmacological inhibition of PI3K with LY294002 abrogated the protective effects of HT. Together, these results suggested that the potential benefits of HT in rats with myocardial I/R injury were likely mediated by the activation of PI3K/Akt pathways. GSK3*β* is an important active enzyme downstream of Akt, which can be phosphorylated by Akt to maintain an inactivated state [29]. The inactivation of GSK3*β* protects against organ ischemic injury, oxidative stress, and apoptosis [30]. In our study, we found an elevated level of phosphorylated GSK3*β* in the myocardium of HT-treated rats, which correlated with reduced cardiac infarction size, decreased cardiomyocyte apoptosis, and preserved heart function. These results further confirm a pivotal role of PI3K/Akt pathways in the potential therapeutic effect of HT. Moreover, it could be concluded that elevated myocardial PI3K/Akt signaling and subsequent increased phosphorylation of GSK3*β* may play important roles in the cardioprotective effects of HT.

Our study has limitations that should be noted when interpreting the results and designing the future studies. First, we performed the study on rat hearts, which are obviously different from human hearts, and further studies are needed to evaluate the clinical use of HT in patients with coronary heart disease. Second, other signaling elements involved in the HT-induced myocardial protective effects need to be identified. Third, long-term studies with adequate sample sizes should be carried out to evaluate whether HT can favorably affect the survival of the I/R model rats. Moreover, the levels of HT and its metabolites in blood and heart were not measured.

In conclusion, the results of our study demonstrated that HT preserved cardiac function by reducing oxidative stress, myocardial infarct size, and cardiomyocyte apoptosis in an* in vivo* model of myocardial I/R injury. Moreover, activation of the PI3K/Akt/GSK3*β* pathway may play a key role in the protective effects of HT.
